# Concomitant L248V With E225V Mutation in the BCR-ABL Gene Associated With Rapid Chronic Myeloid Leukemia Lymphoid Blast Crisis

**DOI:** 10.7759/cureus.58972

**Published:** 2024-04-25

**Authors:** Songphol Tungjitviboonkun, Pawitthorn Wachirapornpruet, Sorrawit Unsuwan

**Affiliations:** 1 Department of Medicine, Sirindhorn Hospital, Bangkok, THA; 2 Department of Medicine, Chulalongkorn University, Bangkok, THA

**Keywords:** all, bcr-abl mutation, blast crisis, imatinib, cml, chronic myeloid leukemia

## Abstract

Chronic myeloid leukemia (CML) is a myeloproliferative neoplasm characterized by the presence of the Philadelphia chromosome (Ph), resulting from the t(9;22)(q34;q11.2) translocation. Imatinib, a tyrosine kinase inhibitor (TKI), has revolutionized the treatment of CML. However, despite the initial response, some patients may progress to an advanced stage, such as a blast crisis.

We report a 40-year-old female who presented with CML chronic phase (CP) taking imatinib 400 mg/day and achieved a complete hematological response (CHR) after one month of treatment. She achieved a suboptimal response in the third month (BCR-ABL positive 10.29% IS). However, five months into therapy, she developed a sudden lymphoid blast crisis with chromosomal aberrations involving chromosomes 10 and 12. Molecular analysis detected concomitant L248V with partial exon 4 deletion and E225V mutations within the BCR-ABL1 fusion gene. The patient received intensive chemotherapy and dasatinib.

We report the first case of concomitant mutation of L248V with partial exon 4 deletion and E255V on BCR-ABL1 gene mutation, which contributes to a sudden precursor B-cell lymphoid blast crisis.

## Introduction

This article was previously posted to the researchsquare preprint server on March 26, 2024.

Chronic myeloid leukemia (CML) is a clonal myeloproliferative disorder characterized by the unregulated proliferation of myeloid precursor cells within the bone marrow. This leads to the accumulation of abnormal white blood cells, primarily granulocytes, in the peripheral blood and bone marrow. The disease is driven by the BCR-ABL1 chimeric gene product that codes for a constitutively active tyrosine kinase, resulting from a reciprocal balanced translocation between the long arms of chromosomes 9 and 22, t(9;22)(q34.1;q11.2), known as the Philadelphia chromosome (Ph) [1].

CML accounts for 15% to 20% of adult leukemia cases. The worldwide incidence is approximately 0.6 to 2.0 cases per 100000 persons, which are more common in males than females, with ratios ranging between 1.3 and 1.8 [2].

According to the European LeukemiaNet (ELN) definition, CML is categorized into different stages based on the progression of the disease, comprising the chronic phase (CP), accelerated phase, and blast phase [3]. The CP usually lasts several years. The accelerated phase lasts four to six months. The blast phase, terminal phase of CML, lasts only a few months [4,5]. The majority of CML cases (>90%) are diagnosed in the CP, while a minority (2.2%) may present with a de novo blast crisis [6]. Patients with blast transformation from CP or accelerated phase can either be myeloid or lymphoid blast crisis. Lymphoid blast crisis accounts for around 30% of CML blast crisis cases [7].

The first-line treatment is a tyrosine kinase inhibitor (TKI). A short course of hydroxyurea may be given in symptomatic patients with high white blood cell or platelet counts while molecular and cytogenetic confirmation of the CML diagnosis is pending. Currently, four FDA-approved TKIs that are commercially available to use as a first-line treatment for CP CML are first-generation imatinib and second-generation dasatinib, nilotinib, and bosutinib.

It is essential to regularly monitor the patient's response to TKI drug therapy in CML to assess the response. The ELN has defined treatment milestones based on the quantity of BCR-ABL1 during treatment [3]. Following imatinib treatment, early molecular response rates at three months (BCR-ABL1 ≤10% IS) range between 60% and 80%. At one and five years, major molecular response (MMR) rates range between 20-59% and 60-80%, respectively [8-10].

Sudden blast crisis (SBC) is categorized as a rapid onset of blast crisis after a documented optimal response to TKI and within three months of a normal complete blood count. Incidence was 0.7% of CML CP patients who were treated with imatinib [11]. Though SBC is rare during the TKI therapy era, it was reported in a patient who, previously achieved MMR, discontinues TKI due to restricted access to health services during the COVID-19 pandemic [12].

Although persistent expression of BCR-ABL leads to genomic instability, there is a report that deletions of the derivative chromosome 9 in CML can lead to rapid progression to blast crisis by loss of one or more tumor suppressor genes(TSG) [13]. Chromosomal abnormalities involving chromosomes 8, 17, 19, and 22 were reported, whether they were associated with genomic stability, with duplication of the Ph chromosome or trisomy 8 being the most frequent in CML blast crisis [14].

There are two reports of L248V BCR-ABL mutation in imatinib-resistant CML patients [15]. Both cases developed disease progression between 15 to 17 months. While the E255V mutation reported in Korea was a relatively more aggressive clinical course in just three months, the patient developed to an accelerated phase, not a blast crisis [16].

This report presents a CML sudden lymphoid blast crisis from concomitant L248V with partial exon deletion and E255V mutation developed in a patient who previously responded to imatinib and had good compliance with medication.

## Case presentation

A 40-year-old female with no known underlying diseases presented to the hospital with a two-day history of high-grade fever. Upon examination, her temperature was 36.6°C, her blood pressure measured 130/80 mmHg, and her respiratory rate was 20/min. A general examination revealed no pallor. Abdominal examination indicated mild hepatomegaly and splenomegaly 4 FB BLCM.

Initial investigations revealed Hb of 11.3 g/dL, WBC of 92790 cells/mm^3^, neutrophils of 67% lymphocytes of 14%, monocyte of 3%, eosinophil of 3%, basophil of 3%, band form of 2%, blast of 1%, metamyelocyte of 3%, promyelocyte of 3%, and platelet count of 737000/mm^3^. BCR-ABL by reverse transcription PCR (RT-PCR) from blood was positive >55% IS. Bone marrow biopsy showed 95% cellularity, marked myeloid predominance, and increased megakaryocytes. Bone marrow aspiration revealed markedly hypercellular marrow, myeloid:erythroid ratio of 10:1, and myeloblast of 2%. Chromosome study showed 46, XX,t(9;22)(q34;q11.2)(20). She was diagnosed with the CML CP and intermediate risk Sokal score. Imatinib 400 mg oral per day was started in December 2023.

Management and outcome

One month after starting imatinib, the patient achieved complete hematological response (CHR), with Hb of 10.0 g/dL, WBC of 2760/mm^3^ with normal differential counts, and platelet count of 288,000/mm^3^. Physical examination showed no hepatomegaly and no splenomegaly. She reported good compliance with imatinib.

Three months after starting imatinib, CBC was normal with Hb of 10.7 g/dL, WBC of 7370/mm^3^, neutrophils of 66%, lymphocytes of 28%, eosinophil of 1%, monocyte of 3%, and platelet of 268000/mm^3^. BCR-ABL was positive 10.29% IS, compatible with suboptimal response, according to ELN definition. After discussing it with the patient, she decided to continue with imatinib 400 mg/day.

**Figure 1:**
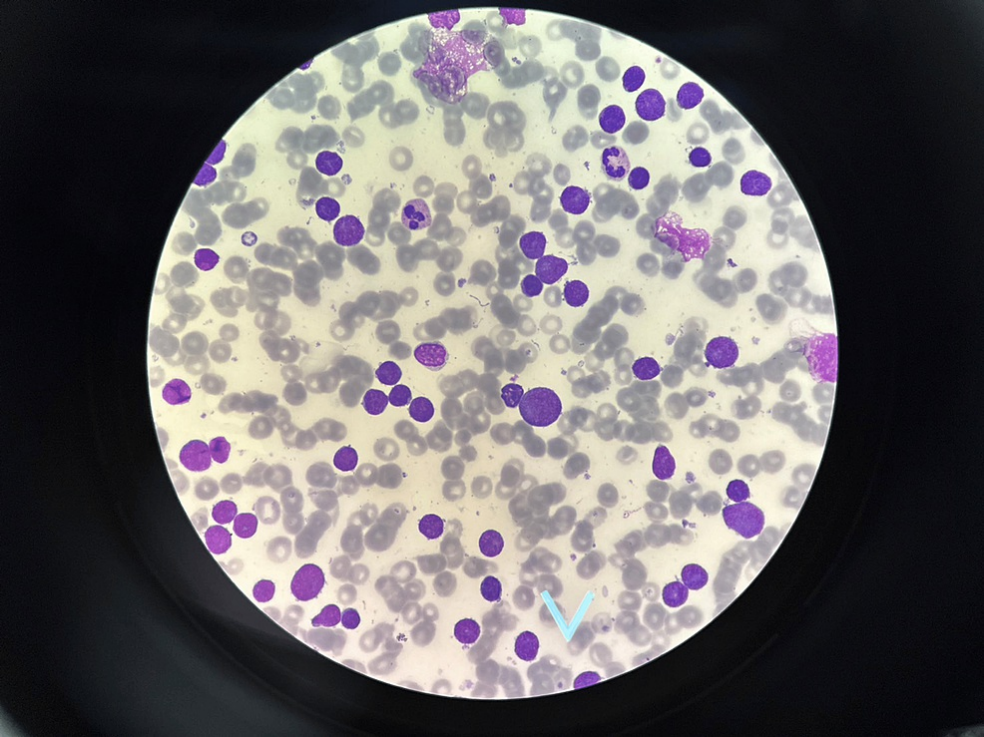
Peripheral blood smear Lymphoblast more than 90% of the white blood cells. Platelets were decreased from normal in this oil field (x100).

**Figure 2:**
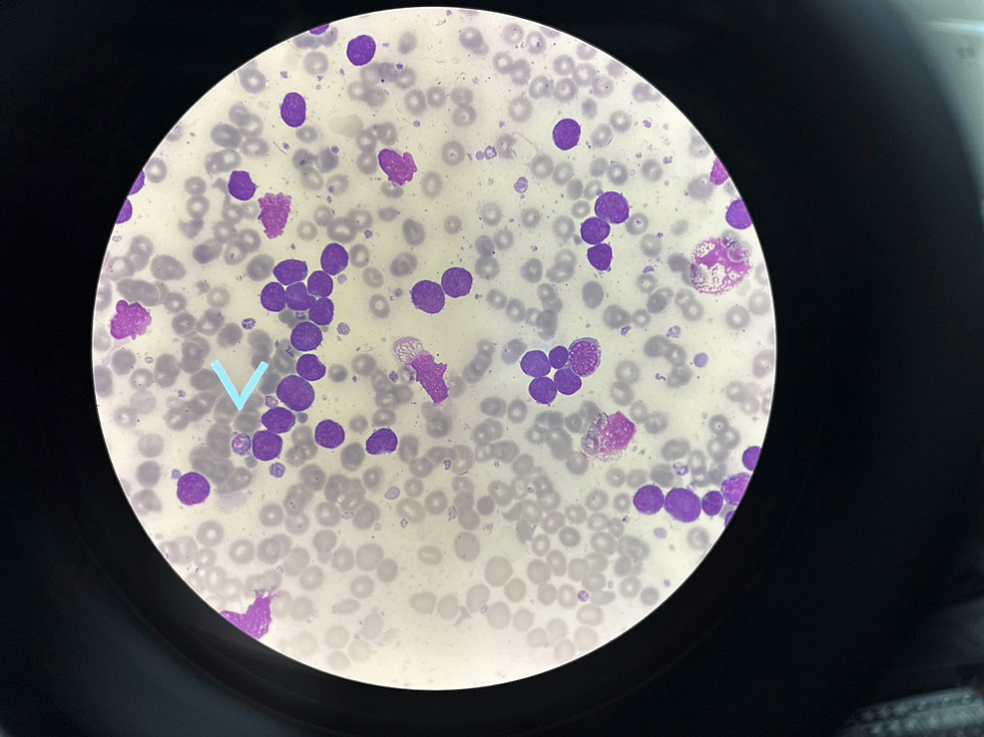
Peripheral blood smear This area seen a group of lymphoblast with decreased platelet count in this oil field (x100).

Five months after starting imatinib, the patient developed fatigue, dyspnea, and gum bleeding. Physical examination revealed a body temperature of 36.5 C, blood pressure of 167/94 mmHg, pulse rate of 97/min, respiratory rate of 20/min, mild hepatomegaly, and splenomegaly of 2 FB BLCM. Her CBC revealed Hb of 10.2, Hct of 31.6%, WBC of 269490/mm^3^ with 94% of lymphoblast, and platelet count of 52000/mm^3^. Peripheral blood smear showed markedly increased lymphoblasts (Figures 1, 2). Bone marrow biopsy showed small foci of atypical large cells with blastic nuclear appearance infiltration. Bone marrow aspiration revealed markedly hypercellular marrow, with 90% lymphoblast. Flow cytometry showed CD10+, CD19+, CD34+, HLA-DR+, and TdT+ blasts with aberrant CD33 expression compatible with precursor B acute lymphoblastic leukemia (ALL). Chromosome study was 46,XX,t(9;22)(q34;q11.2),del(12)(q22q24.1)(8)/ 46,XX,t(9;22)(q34;q11.2),ins(10;12)(q22;q22q24.1)(3). BCR-ABL1 protein p210 (b2a2) was positive and p190 was negative. BCR-ABL mutation gene assay with peripheral blood sample (by RT-polymerase chain reaction) detected L248V with partial exon 4 deletion and E225V mutation. She was diagnosed with CML with lymphoid blast crisis.

The patient received a pediatric-adapted Ph-positive-ALL treatment protocol due to being 40 years old. She received vincristine (2 mg/week intravenously for four doses), doxorubicin (30 mg/m^2^ weekly for three doses), prednisolone (60 mg/m^2^ for 28 days), and asparaginase (5,000 U/m^2^ for 10 days), together with switching the TKI from imatinib to dasatinib 140 mg/day. Due to intensive chemotherapy, she developed two episodes of *Candida tropicalis* septicemia and *Stenotrophomonas* septicemia. She died from septic shock, nonresponsive to multiple antibiotics seven months after the initial CML diagnosis.

## Discussion

Imatinib is one FDA-approved, first-line treatment for CP CML. Although the patient has responded to imatinib by achieving CHR before, mutations within the tyrosine kinase domain of the ABL gene are a significant contributor to resistance against TKIs in individuals with CML. These mutations are observed in a substantial portion of CML patients who experience resistance, with prevalence ranging from 30% to 90%, depending on the studies [17,18]. These mutations encompass over 40 distinct amino acid changes, each imparting varying degrees of resistance to imatinib, a commonly used TKI [17].

Based on current knowledge, it is not advisable to routinely conduct mutation screening unless there are specific reasons to do so, such as a loss of treatment effectiveness [19]. Identifying mutations in the ABL gene during the CP of CML treatment may potentially improve treatment outcomes. Nevertheless, it is important to note that regular ABL mutation screening is not generally recommended for CML patients [20].

In this study, we found a concomitant mutation of L248V with partial exon 4 deletion and E255V on the BCR-ABL1 gene mutation and chromosomal aberrations involving chromosomes 10 and 12, which contributes to not only resistance to TKIs but also a sudden lymphoid blast crisis.

## Conclusions

This case underscores the complexity and variability in the genetic landscape of CML, emphasizing the importance of monitoring and understanding mutations in the BCR-ABL1 gene and associated chromosomal aberrations. The presence of the L248V with partial exon 4 deletion and E255V variants highlights the evolving nature of resistance mechanisms that can emerge during treatment with TKIs like imatinib. These mutations not only compromise the efficacy of targeted therapies but also pose a risk for disease progression, as evidenced by the sudden transition to a lymphoid blast crisis in this patient.

Furthermore, the identification of chromosomal aberrations involving chromosomes 10 and 12 adds another layer of complexity to the disease course and treatment response. These genetic alterations may interact synergistically with BCR-ABL1 mutations, exacerbating resistance and contributing to the aggressive transformation of the disease. While routine mutation screening is not generally recommended for all CML patients, this case underscores the importance of targeted genetic testing in cases where treatment effectiveness is compromised or when disease progression is suspected. Understanding the specific mutations and chromosomal aberrations driving resistance and disease progression can inform treatment decisions, potentially guiding the selection of alternative therapies and improving clinical outcomes for patients with CML.
